# Preclinical Magnetic Resonance Imaging and Spectroscopy Studies of Memory, Aging, and Cognitive Decline

**DOI:** 10.3389/fnagi.2016.00158

**Published:** 2016-06-29

**Authors:** Marcelo Febo, Thomas C. Foster

**Affiliations:** ^1^Department of Psychiatry, William L. and Evelyn F. McKnight Brain Institute, University of FloridaGainesville, FL, USA; ^2^Department of Neuroscience, William L. and Evelyn F. McKnight Brain Institute, University of FloridaGainesville, FL, USA

**Keywords:** fMRI, DTI, magnetic resonance spectroscopy, hippocampus, memory, preclinical MRI, aging neuroscience

## Abstract

Neuroimaging provides for non-invasive evaluation of brain structure and activity and has been employed to suggest possible mechanisms for cognitive aging in humans. However, these imaging procedures have limits in terms of defining cellular and molecular mechanisms. In contrast, investigations of cognitive aging in animal models have mostly utilized techniques that have offered insight on synaptic, cellular, genetic, and epigenetic mechanisms affecting memory. Studies employing magnetic resonance imaging and spectroscopy (MRI and MRS, respectively) in animal models have emerged as an integrative set of techniques bridging localized cellular/molecular phenomenon and broader *in vivo* neural network alterations. MRI methods are remarkably suited to longitudinal tracking of cognitive function over extended periods permitting examination of the trajectory of structural or activity related changes. Combined with molecular and electrophysiological tools to selectively drive activity within specific brain regions, recent studies have begun to unlock the meaning of fMRI signals in terms of the role of neural plasticity and types of neural activity that generate the signals. The techniques provide a unique opportunity to causally determine how memory-relevant synaptic activity is processed and how memories may be distributed or reconsolidated over time. The present review summarizes research employing animal MRI and MRS in the study of brain function, structure, and biochemistry, with a particular focus on age-related cognitive decline.

## Introduction

Among the various techniques in neuroscience, magnetic resonance imaging and spectroscopy are uniquely suited for longitudinal measurements; providing in-depth assessments of neural activity, tissue microstructural organization, and chemistry in the aging brain. Functional and diffusion magnetic resonance imaging (fMRI and dMRI, respectively) are among the most promising MRI modalities that may be used to investigate the relationship between regional changes in neural activity and structural connectivity. These neuroimaging methods have been employed in aging humans to suggest that variability in the decline of several cognitive processes results from changes in defined neural circuits (O’sullivan et al., 2001; Gunning-Dixon and Raz, 2003; Salat et al., 2005; Dennis et al., 2008; Kaufmann et al., 2008; Duverne et al., 2009; Wang et al., 2009; Lighthall et al., 2014). However, cellular and synaptic mechanisms underlying regional differences in vulnerability to aging are difficult to assess in human subjects. Thus, the utility of fMRI and dMRI in studying functional and neuroanatomical correlates of the human aging brain is strengthened by animal studies that combine imaging with invasive assessments. Animal imaging approaches combining fMRI with electrophysiological recordings, direct electrical stimulation and/or optogenetic modulation of neuronal activity, may bring us closer to characterizing links between neural activity and memory formation, both in healthy aging and with cognitive impairment. Other animal imaging methods not widely used in human subjects, such as pharmacological MRI (**Box 1**), may be used to discern specific drug effects on BOLD activity in memory networks. Functional and anatomical imaging techniques find strong complementation with *in vivo* magnetic resonance spectroscopy (MRS), which describes biochemical correlates in memory regions.

**Box 1. *Pharmacological MRI* is a term used to describe the use of functional magnetic resonance imaging modalities to measure the BOLD response to neuropharmacologically active compounds (Chen et al., 1999; Salmeron and Stein, 2002).**

Studies typically employ BOLD imaging, however, the term is inclusive of arterial spin labeling, iron contrast-based cerebral blood volume measurements, and manganese enhanced MRI studies designed to screen brain activity in response to CNS drugs.

The present article provides an overview of animal studies that use fMRI, dMRI, and MRS to assess functional, structural, and chemical characteristics in brain areas involved in learning and memory. Rather than providing an extensive overview of the full breadth of the animal imaging literature, the review focuses on studies that are particularly relevant to normal aging animal models, and on imaging and spectroscopy studies of temporal lobe, prefrontal cortical and striatal circuits. The medial temporal lobe episodic memory system and a prefrontal cortex and striatal executive function system are highly vulnerable to changes in structure and activity associated with cognitive decline in humans, monkeys, rats, and mice. Furthermore, there is a rich repertoire of behavioral paradigms that can be applied to study of age-related decline in memory and executive function across species (Moss et al., 2007; Nagahara et al., 2010; Zeamer et al., 2011; Alexander et al., 2012; Bizon et al., 2012; Foster et al., 2012; Holden and Gilbert, 2012; Roberson et al., 2012; Crystal and Wilson, 2015). Monkeys may have an advantage for behaviors that depend on the higher complexity of the cortex. In this regard, the anatomy of the prefrontal cortex in Old world primates is more analogous to that of humans. However, aged non-human primates may exhibit extensive neuronal loss in the prefrontal cortex, which is not evident in aging humans or other animal models (Smith et al., 2004; Burke and Barnes, 2006). Furthermore, while comparative studies have identified similarities in connectivity for auditory and visual system pathways, some connections involving the prefrontal cortex may be absent in non-human primates (Rilling, 2014), which may underlie differences in behaviors between humans and non-human primates (Stoet and Snyder, 2003). In contrast, rodents are a common model of “normal” cognitive aging, particularly for studies seeking to understand cellular and molecular mechanisms underlying age-related changes in brain structure and function. We will attempt to offer interpretations on the summarized literature and discuss how the imaging findings might be reconciled with what is known on the synaptic circuitry and mechanisms of learning and memory.

## Emerging Approaches to Drive and Record from Memory Circuits During fMRI

Findings from fMRI experiments are perhaps the most intriguing among preclinical imaging studies because of the potential of resolving functional changes involving hippocampal and prefrontal circuits during specific stages of memory formation and reconsolidation. A major question concerns the underlying neuronal activity that generates the BOLD signal. Is the signal related to region specific neuronal discharge activity or does it reflect synaptic activity associated with afferent input and local circuits? Several studies have examined neuronal discharge activity in behaving animals. Based on visual stimulation induced changes in BOLD signal in humans and neuronal discharge activity in monkeys it was suggested that the BOLD signal is representative of neuronal firing rate (Heeger et al., 2000; Rees et al., 2000). However, in both rats and non-human primates BOLD fMRI signals correlate more closely with local field potentials (LFPs) than with multi-unit activity (MUA; Logothetis et al., 2001), although recent work in rats indicates that cerebral blood flow (CBF) correlates better with LFPs than do BOLD signals or cerebral blood volume (CBV; Herman et al., 2013). The LFPs represent relatively slow changes in membrane depolarization and hyperpolarization due to afferent input and local circuit synaptic activity. Thus, the BOLD response is largely associated with local neuronal processing of synaptic inputs, as well as excitatory and inhibitory synaptic activity of the local circuit, rather than the consequent neuronal discharge activity which represents the output computation (Logothetis and Wandell, 2004). Thus, it is possible that neuromodulatory influences that inhibit the discharge activity of principle cells can increase the BOLD response, while increased discharge activity due to GABA antagonist may not alter the BOLD response (Thomsen et al., 2004). In this regard, the BOLD signal will differ across regions due to the local excitatory/inhibitory configuration of the circuit.

### Functional Imaging of Hippocampal Networks

Taking advantage of the well-defined synaptic circuitry of the hippocampal formation, Angenstein et al. (2007, 2009) have utilized direct current stimulation and neural recordings across a series of studies to determine the relationship between local field activity and the BOLD signal, particularly in relation to its evoked spatial and temporal properties in hippocampus (Tiede et al., 2012; Angenstein, 2014; Scherf and Angenstein, 2015). Electrical stimulation of afferents and recording in specific hippocampal regions allowed this group to control input activity to the dorsal CA1/dentate region, where BOLD signals were measured. Using this technique they determined important properties of hippocampal BOLD responses in relation to the neuronal activity driving this signal. For BOLD response originating in the dentate gyrus, it seems that afferent synaptic activity of the perforant path correlates better with BOLD responses rather than the discharge response of the population of granule cells (Angenstein et al., 2007). Furthermore, the propagation of BOLD activity across interconnected hippocampal subregions is influenced by the internal processing dynamics and synaptic plasticity in this region (Angenstein et al., 2009). The induction of long-term potentiation (LTP) requires activation of *N*-methyl-D-aspartate (NMDA) receptors (Foster, 2012), thus treatment with an NMDA receptor blocker (MK801) prior to afferent stimulation blocks hippocampal network activity (Tiede et al., 2012). Hence, increased BOLD signal changes associated with the induction of LTP suggests that memory-related changes in neural activity are measurable with fMRI (Canals et al., 2008; Angenstein et al., 2013).

**FIGURE 1:**
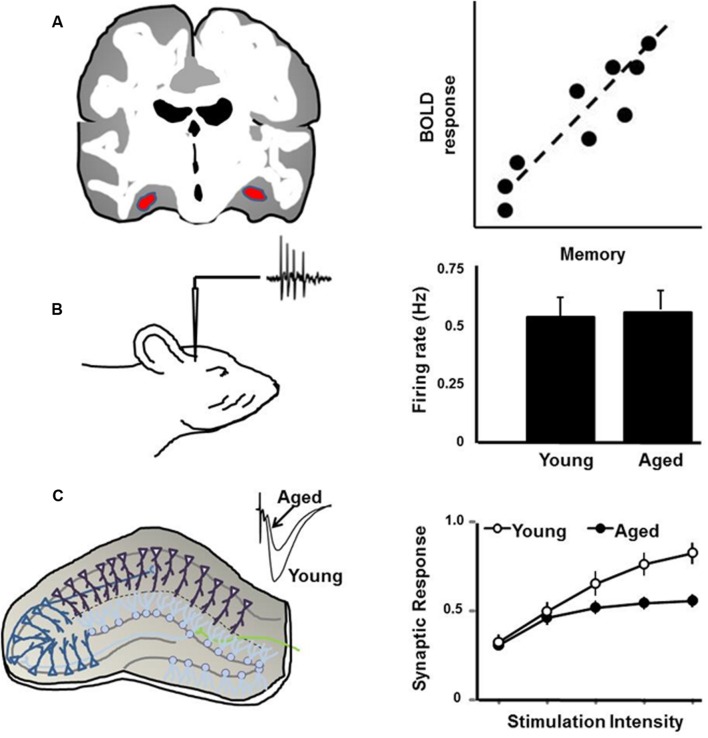
**Schematic illustration of (A) the BOLD signal, which is positively correlated with memory, such that impaired memory is associated with reduced BOLD activity in the hippocampus. (B)** Discharge activity of hippocampal cells in response to environmental stimuli is not related to age or memory. Rather, cognitive impairment is associated with reduced ability to modify discharge activity. **(C)** Synaptic transmission and synaptic plasticity in the hippocampal slice is reduced with age, particularly in memory-impaired animals.

Here is where animal-imaging studies may provide key insights in the regulation of neural activity during memory formation and recalling specific events, as reported in human subjects (Ranganath et al., 2004; Flegal et al., 2014). Parahippocampal areas of normal healthy human subjects show greater BOLD responses during correct recall of events than with incorrect recall (Eldridge et al., 2000). In awake Rhesus macaques, correct recall of events on a serial probe recognition task was associated with increased BOLD in caudal entorhinal cortex, perirhinal cortex and hippocampus (Miyamoto et al., 2014). While this temporal lobe network in primates reflects early consolidation phases, the intraparietal sulcus plays a role in long-term retrieval processes (Miyamoto et al., 2013). The idea that synaptic activity mediates the BOLD response has important implications for interpreting the response as it relates to mechanisms for cognitive function during aging (**Figure 1**). Impaired memory encoding and retrieval is associated with decreased BOLD activity in the hippocampus and medial temporal lobe of humans (Daselaar et al., 2003; Morcom et al., 2003; Dennis et al., 2008; Salami et al., 2012; Pudas et al., 2013; Sherman et al., 2015). Conversely, an increase in frontal cortex neural activity is observed in older humans and may relate to performance of executive function tasks (Rosano et al., 2005; Turner and Spreng, 2012; Maillet and Rajah, 2014). Studies in animals suggest that the level of cell discharge activity is not dramatically altered in the hippocampus or prefrontal cortex of older cognitively impaired animals (Oler and Markus, 2000; Burke and Barnes, 2006; Wang et al., 2011; Caetano et al., 2012). Rather, cognitive decline is associated with an inability to modify cell discharge activity. In turn, modifiability of cell discharge activity depends on synaptic plasticity, and thus age-related cognitive decline is associated with a decrease in the strength of excitatory synapses and impaired synaptic plasticity (Luebke et al., 2004; Foster, 2012; Guidi et al., 2015b).

In spite of the aforementioned insights, direct electrical current stimulation presents technical challenges preventing straightforward interpretations that can link these results to human imaging work. Among these is the non-specificity of neuronal groups targeted for stimulation, a lack of control over excitatory versus inhibitory activity, and off-target antidromic activation of afferent inputs to the stimulation site that could hinder clear interpretations of fMRI data. Some of these limitations may be resolved through the use of optogenetics in conjunction with fMRI. Initial studies applying this strategy have focused on hippocampal and prefrontal cortical regions. Therefore, these types of experiments are highly relevant to characterizations of circuit adaptations in memory and normal aging. Following a seminal study using the light sensitive cation channel rhodopsin 2 (ChR2) to drive motor thalamocortical BOLD responses (Lee et al., 2010), Lee and colleagues conducted a similar opto-fMRI study centered on eliciting hippocampal activation (Duffy et al., 2015). Light stimulated excitation of dorsal CA1 pyramidal neurons increased BOLD activation in the hippocampus and its output regions in the medial septum (Duffy et al., 2015). Increasing stimulation frequencies to levels capable of triggering seizure-like after discharges elicited a greater distribution of BOLD activation to contralateral hippocampus, neocortex, and mediodorsal thalamus, an effect closely resembling optogenetically evoked BOLD activation in mouse CA1 circuitry (Takata et al., 2015). Among the implied conceptual benefits of the opto-fMRI studies of hippocampal networks is the potential for measuring how neural activity moves through subregions of hippocampal memory networks. Importantly, future studies are likely to target specific cell groups to characterize hippocampal network activity and how memory and recall mechanisms modify activity through this structure. Studies directed at understanding specific roles for neurotransmitter systems in modulating BOLD activation through their effects on LFPs and MUA are coming to fruition in non-human primates (Rauch et al., 2008; Arsenault et al., 2013; Zaldivar et al., 2014). Applying such targeted cell- and receptor-specific approaches in imaging hippocampal networks is likely to provide powerful insight into effects of aging on hippocampal activity, memory, and cognitive behaviors.

### Functional Imaging of Prefrontal Networks

The prefrontal cortex plays a role in working memory and its role in normal aging is functionally distinct from that of hippocampus and amygdala. Due to the complexity of the prefrontal cortex, in terms of afferents, efferents, and local circuits, optogenetics is essential in order to specify neuronal activation patterns with memory and in aging. Optically exciting output neurons from the prelimbic area of the prefrontal cortex of awake rats has been shown to increased BOLD activation in ventral striatum, other neocortical areas, and the mediodorsal thalamus (Liang et al., 2015). It should be noted that BOLD activity in mediodorsal thalamic nucleus occurs with optogenetic stimulation of the hippocampus and prefrontal cortex. This region is known to be an integral part of anterior thalamic limbic circuitry involved in memory and learning (Aggleton and Brown, 1999; Aggleton et al., 2010). Interestingly, mediodorsal thalamic BOLD activation observed when driving prefrontal neurons of awake rats was not observed in rats under anesthesia. Conversely, hippocampally driven mediodorsal thalamus activation occurred under anesthetized conditions (Duffy et al., 2015). The distinct responsiveness of the mediodorsal thalamus to stimulation of these two brain areas thus appears varies according to the state of consciousness of the animals. It is possible that prefrontal cortex-to-mediodorsal thalamus activation requires an awake state whereas it does not appear to be necessary in hippocampal networks. This brings up interesting possibilities regarding the potential properties of temporal and prefrontal lobe interactions in memory networks. Mediodorsal thalamic neurons project to limbic frontal areas such as the prelimbic, orbital, insular and anterior cingulate regions (Gabbott et al., 2005). Here, they form asymmetric synaptic contacts with layer III pyramidal neurons projecting back to mediodorsal thalamus (Kuroda et al., 2004). Mediodorsal thalamic neurons also synapse onto two types of GABAergic interneurons that modulate both pyramidal cells and GABAergic interneurons, thus offering a potential network controlling and modifying thalamocortical and corticocortical activity (Kuroda et al., 2004). This medial thalamic circuitry also modulates hippocampal-to-prefrontal activity (Floresco and Grace, 2003). Driving hippocampal activity to the prefrontal cortex is modulated by stimulation of mediodorsal thalamus (Floresco and Grace, 2003). Tetanic stimulation of mediodorsal thalamus-to-prefrontal neurons potentiates ventral hippocampal-to-prefrontal activity (Floresco and Grace, 2003). Thus, the hippocampal-prefrontal circuitry shows synaptic plasticity that is under partial control by mediodorsal thalamic neurons. These, and other results, strongly suggest that mediodorsal thalamic neurons regulate the transit of limbic activity to and from frontal cortical and hippocampal networks, and it also offers a pathway that can be targeted for further opto-fMRI studies.

The functional role of the dorsolateral area of the prefrontal cortex in working memory has been characterized using fMRI in humans and neurophysiological recordings in non-human primates (Funahashi et al., 1989; Curtis and D’Esposito, 2003; Nee and D’Esposito, 2016). Working memory tasks that engage this region elicit a BOLD activation pattern that reflects its temporary storage buffer and processing capacity (D’Esposito et al., 1999a; Rypma and D’Esposito, 1999), with a temporal neural activity profile similar to that measured electrophysiologically in non-human primates (Funahashi et al., 1989; Compte et al., 2000). It appears that with aging there is compensatory increased activation in the dorsolateral prefrontal cortex, with reduced BOLD activation in caudal sensory processing structures, such as the temporal and occipital cortices (Gigi et al., 2010; Fakhri et al., 2012). Older adults show greater BOLD activation in this prefrontal region that expands to the contralateral site when compared to young individuals (Cabeza et al., 2004). The greater BOLD activation in older versus young individuals may be related to a compensatory activation of neurons in this region during a working memory task. Interestingly, it was previously shown that the BOLD response to a high load working memory task is higher and more lateralized (to the right hemisphere of the laterodorsal prefrontal cortex) than in a low load working memory condition (Rypma and D’Esposito, 1999). More recent, work in older individuals has shown that this pattern may vary, with high cognitive load eliciting weaker activation (failure to meet demands) and low load activating the region more strongly (compensation for the functional loss; Cappell et al., 2010; Toepper et al., 2014). It is unclear if the expansion is in fact compensation in order to facilitate behavior, or is a sign of decreased specificity, and/or a sign of the impairment. Comparable studies have not been carried out in rats in order to address this matter more directly by manipulation of frontal cortical brain areas (Liang et al., 2015). Therefore, this is an area that would greatly benefit from opto-fMRI studies in rodents. During working memory tasks, the discharge activity of some cells does not increase to the same extent in aged rats (Caetano et al., 2012) and monkeys (Wang et al., 2011). In turn, the shift in discharge activity is thought to results from a shift in the balance of excitatory/inhibitory synaptic activity (Luebke et al., 2004; Guidi et al., 2015b), including the loss of dendritic spines (Dumitriu et al., 2010). If the BOLD expands in older humans, then the decline in discharge activity would not directly explain this. An alternative would be that decreased activity in the region may result in decreased lateral inhibition, permitting increased activity of other regions (thus the expansion of BOLD to other areas). Such a shift in the balance of excitatory/inhibitory synaptic activity could explain the expansion, and is an intriguing target for optogenetic manipulations in aged rats.

In summary, fMRI studies designed to activate the hippo campus of rats reveal that causally driving afferent inputs to the dentate gyrus via the perforant path increases BOLD in this region, and in downstream areas, and this appears to involve dynamic processing of synaptic activity. Variations in BOLD associated with high frequency pattern stimulation are likely due to synaptic plasticity in local circuits. These plasticity mechanisms, which can be engaged during learning or due to pathology such as epilepsy, influence the spread of neural activity to connected regions. Finally, a decrease in synaptic strength or plasticity, or a shift in the balance of excitatory/inhibitory synaptic activity may underlie changes in the BOLD response in association with cognitive aging.

## Imaging Resting State Networks Involved in Memory

Resting state fMRI is becoming a highly valuable strategy for characterizing neural circuits involved in learning and memory, especially when measures of behavioral performance on cognitive tasks are also assessed. Resting state connectivity provides information on intrinsic functional brain organization, which under baseline conditions involves correlated BOLD signals between specific subsets of brain areas (e.g., default, executive, salience networks can be assessed). Similarity in resting state functional connectivity of the hippocampus is observed between humans and animals models, including rodents, rabbits and monkeys (Becerra et al., 2011; Hutchison et al., 2011; Jonckers et al., 2011; Schroeder et al., 2016). Resting state connectivity has been examined in awake marmoset and rhesus monkeys and in anesthetized macaques. Effective connectivity (which estimates directionality of connectivity) between hippocampus and parietal cortex increases during memory retrieval in awake macaques (Miyamoto et al., 2013). Areas of the default mode network (e.g., medial and lateral parietal areas, anterior and posterior cingulate, and medial prefrontal cortex) exhibit decreases in activity during performance of goal-directed and attention-demanding tasks, and show increase functional coupling when the brain is in an “idle” mode (Raichle et al., 2001). This network appears to be active in anesthetized monkeys (Hutchison et al., 2011; Mantini et al., 2011, 2013) and rats (Upadhyay et al., 2011; Lu et al., 2012), and activity is decreased as monkeys attend to external stimuli (Mantini et al., 2011). Macaques and humans have a homologous temporal-parietal resting state network that involves parahippocampal areas, retrosplenial, posterior cingulate, superior temporal gyrus, and posterior parietal cortex, which may be involved in mnemonic processes (Vincent et al., 2010). Some of these areas are also part of the default network and this further strengthens the notion that this system is preserved across species of primates and rodents (Kojima et al., 2009; Lu et al., 2012), although a difference in the role of the striatum within the default system has been reported between human and non-human primates (Kojima et al., 2009).

Functional connectivity networks are increasingly used to assess network-level alterations associated with learning and memory and conditions of impaired cognition including aging. In rats, it has been shown that training-induced improvement in performance on a Morris water maze (MWM) task is associated with increased connectivity within hippocampal regions and between the hippocampus and other memory associated regions such as the septum, retrosplenial cortex, entorhinal cortex, and task associate regions such as the visual and motor cortices and thalamus (Nasrallah et al., 2016). The increased connectivity between these regions was again reduced 7 days after the last MWM session, suggesting a waning of memory associated network BOLD activity (Nasrallah et al., 2016). In humans, resting state connectivity is reduced with age (Achard and Bullmore, 2007; Andrews-Hanna et al., 2007; Damoiseaux et al., 2008; Wu et al., 2011; Dennis and Thompson, 2014; Song et al., 2014; Ferreira et al., 2015; Huang et al., 2015; Li et al., 2015; Scheinost et al., 2015; La et al., 2016). Thus, one possibility is that a decrease in resting state connectivity is an indication of impaired memory formation or consolidation during aging. In a study that examined changes in resting state connectivity, older rats exhibited a postoperative impairment in cognition associated with decreased resting state connectivity, which recovered over time (Xie et al., 2013). In contrast, results obtained in middle age non-human primates showed increased connectivity strength between hippocampus and neocortical areas in animals with low memory performance scores (Koo et al., 2013). These animals showed reduced white matter integrity, suggesting that loss of memory performance with aging is associated with increased functional connectivity to compensate for structural white matter losses (Koo et al., 2013). Similarly, it should be noted that patients diagnosed with mild cognitive impairment exhibited increased connectivity between hippocampal and prefrontal regions, which the authors suggest is a result of a maladaptive reorganization of the brain (Gardini et al., 2015). It is thus possible that increased hippocampal functional connectivity reflects compensatory increases in neuronal activity in temporal lobe and neocortical networks of middle aged individuals, or individuals with milder forms of cognitive impairment. Although not yet determined, such compensatory neuronal activity might fail at later ages, or with the progression of senescent synaptic function with more advanced age and/or dementia.

In sum, experimental paradigms recruiting memory systems in normal aging may modify patterns of resting state functional connectivity across specific functional networks involving default mode and temporal lobe areas that are preserved across species (hippocampal areas in case of rats). While there is an extensive literature linking aspects of Alzheimer’s disease and other forms of neurodegenerative dementia’s to alterations in these networks, normal aging connectivity patterns need further investigation. Of note is the fact that functional connectivity analysis is based on statistical correlation methods and, as a result, limits the establishment of causal links to cellular and synaptic mechanisms. In spite of this limitation, future animal imaging studies should further define links between neuronal aging mechanisms and distinct functional connectivity patterns associated with impaired cognitive function.

## Functional Imaging and Neurovascular Coupling Deficits in Cognitive Aging

Neurovascular coupling is a critical aspect of BOLD fMRI that can be impacted by cellular and molecular events altered in aging, especially as it relates to vascular mechanisms (D’Esposito et al., 1999b). This in turn could directly affect cognitive performance, even in the absence of data indicating impairments in synaptic plasticity. Cerebral metabolic rates for oxygen, arterial perfusion and blood volume changes contribute to the BOLD effect and may all be independently influenced by an aging cerebrovasculature (Mehagnoul-Schipper et al., 2002). Also, supporting cells, such as astrocytes, and the expression of vasoactive molecules, which play an important role in neurovascular coupling (Takano et al., 2006; Drake and Iadecola, 2007) may also be affected by aging mechanisms and in turn affect functional MRI results. Functional MRI alterations in the aging brain may, therefore, be influenced not only by changes in synaptic activity and strength, but may also occur as a result of changes in neurovascular coupling.

Aged rats show lower oxyhemoglobin, CBF and percent increases in BOLD signal in cortex in response to hypercapnia than young rats (Desjardins et al., 2014). These perfusion deficits worsen with age, and even more so with hypertension (Lee et al., 2011, 2014). Interestingly, deficits in response to hypercapnic challenge show a linear relationship with mild cognitive impairments in aged rats and are thought to be predictive of reduced performance on cognitive tasks (Mitschelen et al., 2009). Direct effects of aging on neurovascular uncoupling may contribute to reductions in cognitive performance, even in the absence of a change in synaptic function. Inhibiting the vasoactive signaling molecules cyclooxygenase-2, epoxygenase, and nitric oxide synthase (NOS) reduced CBF in response to whisker barrel stimulation. Reduced CBF was in turn associated with reduced performance in Y- and T-maze tasks and object recognition in the absence of altered synaptic strength (Tarantini et al., 2015). Cerebrovascular insufficiency has been shown to be associated with reduced performance on a MWM task, increased CA1 neuron damage, increased glial acidic fibrillary protein (GFAP) expression, reduced hippocampal blood flow, and increased ^31^P-phosphomonoester, which may be an indicator of altered membrane phospholipid turnover rates (de la Torre et al., 1992). The results suggest that vascular impairments with age might lead to blunted BOLD signal responses compared to young adults and contribute to impaired cognition.

In aged animals the BOLD response is linked to several biological markers that are thought to contribute to cognitive deficits. Transcriptional profiling in vulnerable brain regions has revealed a relationship between age, cognitive function and gene expression (Prolla, 2002; Blalock et al., 2003; Loerch et al., 2008; Zeier et al., 2011). In general, aging is associated with increased expression of genes associated with the immune response and a decrease in expression of genes linked to synaptic connectivity and neural activity. Gene changes are relatively region-specific and suggest regional vulnerability to aging (Wang and Michaelis, 2010; Zeier et al., 2011). The regional specificity for an altered BOLD response suggests that the blunted BOLD signal may be due to local changes in synaptic function, metabolism, and neuroinflammation associated with these gene expression changes (Blau et al., 2012; Moreno et al., 2012; Sanganahalli et al., 2013). In support of this notion, several recent studies have combined gene manipulations with neuroimaging to understand the relationship between transcriptional markers of aging or neurodegenerative disease and the progression brain changes (Song et al., 2004; Maheswaran et al., 2009; Moreno et al., 2012; Lewandowski et al., 2013; Pavlopoulos et al., 2013; Zerbi et al., 2014; Micotti et al., 2015; Sevgi et al., 2015). Mutation or absence of the cholesterol transporter protein apolipoprotein-𝜀 (ApoE) is associated with deficits in functional connectivity and in CBF in the mouse hippocampus (Zerbi et al., 2014). The functional impairments are associated with increased mean diffusivity, which in turn are linked to synaptic loss and presence of pro-inflammatory cells in the region. In one study, a decline in the expression of histone binding protein RbAp48 was observed specific to the dentate gyrus of humans and mice over the course of aging (Pavlopoulos et al., 2013). Expression of a dominant-negative inhibitor of RbAp48 resulted in impaired memory and a decrease in BOLD activity within the dentate gyrus suggesting that altered histone regulation underlies cognitive impairment and/or decreased BOLD activity. The idea that age-related cognitive impairments are associated with a decrease in activation of the dentate gyrus is supported by work in a primate model of aging showing reduced CBV, which was associated with reduced expression of the neural activity marker *Arc* (Small et al., 2004). Thus, the above-cited studies appear to arrive at a consensus that age related reductions in CBF are particularly robust in the dentate gyrus. This represents a promising direction for preclinical imaging research.

## *In Vivo* Hippocampal and Cortical Volumetric Changes Associated with Aging

A reduction in synaptic connectivity in the hippocampus with aging may consequently produce atrophy of this structure, impair memory functions, and this may explain not only impaired BOLD fMRI and vascular changes, but also volumetric changes in this region. There is currently a debate as to whether cognitive aging is associated with a decline in hippocampal volume in humans (Jernigan et al., 1991; Golomb et al., 1993; Sullivan et al., 1995; Raz et al., 2000), non-human primates (Shamy et al., 2006; Willette et al., 2013), and dogs (Su et al., 1998; Tapp et al., 2004; Kimotsuki et al., 2005). In aged Rhesus macaques, spatial memory was not associated with the size of the hippocampus; although, expression of the synaptic marker synaptophysin was reduced in animals with impaired memory (Haley et al., 2012). In contrast, another study indicated that gray matter areas of the prefrontal and temporal cortices of macaques show age related reductions in volume accompanied by reduced performance on delayed non-match to sample task (Alexander et al., 2008; Wisco et al., 2008). Synaptic proteins and mRNA levels, and hippocampal volumes, decline in aged-memory impaired rats, with volumes lower in aged and middle age rats compared to young rats (Smith et al., 2000; Blalock et al., 2003; Driscoll et al., 2006). The decline in hippocampal volume correlated with reduced performance on a MWM task. Similarly, transgenic mice expressing ApoE4 show greater aged related reductions in hippocampal and cortical volumes, and also suffer greater cognitive deficits than wild-type mice (Yin et al., 2011). The reduced hippocampal volume is associated with increased microglial marker iba1 and tumor necrosis factor α, suggesting a role for neuroinflammation. Finally, aged lemurs that performed poorly on shifting and discrimination tasks also show significant volumetric reductions in caudate-putamen, hippocampus, septum, and temporal, occipital and cingulate cortices (Picq et al., 2012). Volumetric reductions may be influenced by co-occurring conditions affecting the aging population. For instance, using a heart failure model, Suzuki et al. (2015) showed a significant reduction in gray matter volume in rats with coronary ligation. There is also promising evidence suggesting that physical activity may ameliorate reductions in cortical and hippocampal volumes (Fuss et al., 2014; Mariotti et al., 2014; Sumiyoshi et al., 2014).

Volumetric changes can be linked to functional changes through the use of manganese enhanced MRI (MEMRI) in animal studies (Koretsky and Silva, 2004). The paramagnetic manganese ion (Mn^2+^) enters voltage dependent Ca^2+^ channels (VDCC), which are pervasively present on synapses across the brain. It is therefore used as a surrogate marker of synaptic activity during baseline conditions and following chronic disease states, memory tasks, or drug stimulation (Pautler et al., 2003; Pautler, 2004). Intra-synaptic sequestering and transsynaptic transport allows for the measurement of neural circuit activity-associated increases in signal intensity in high resolution T_1_ weighted images. A popular application of MEMRI is to quantify rates of signal intensity change in major fiber pathways in order to indirectly estimate *in vivo* brain axonal transport rates (Bearer et al., 2007; Smith et al., 2007, 2010, 2011; Kim et al., 2011). Using this methodology, Cross et al. (2008) showed a significant decline in olfactory pathway transport rates in aged vs. young and middle aged rats. This has been demonstrated as well in mouse models of amyloidosis, tauopathy, and neurodegeneration (Serrano et al., 2008; Majid et al., 2014).

Interestingly, given its mechanism involving VDCC uptake, this imaging strategy can provide an indication of Ca^2+^ regulation (Lu et al., 2007; Berkowitz et al., 2014; Groschel et al., 2014). Dysregulation of Ca^2+^ during aging is thought to underlie changes in cell excitability (Landfield, 1988; Disterhoft et al., 1993; Foster, 2007; Thibault et al., 2007; Kumar et al., 2009; Oh and Disterhoft, 2010) and the senescence of synaptic function (Foster and Norris, 1997; Kumar et al., 2009; Foster, 2012). The MEMRI technique has been employed to demonstrate increased Ca^2+^ of sensory systems associated with an age-related impairment of sensory processing (Bissig et al., 2013; Groschel et al., 2014). For example, 13- to 18-month-old mice with significant hearing loss show greater accumulation of Mn^2+^ signal in auditory networks and the hippocampus relative to 3-month-old mice (Groschel et al., 2014). An increase in MEMRI signal intensity in the pyramidal cell layer of the CA1 is also observed in 6- to 18-month-old rats (Bissig and Berkowitz, 2014). However, it is unclear if the changes represent accumulation in active neurons, active synapses, or glial cells (Nairismagi et al., 2006; Hsu et al., 2007; Immonen et al., 2008; Eschenko et al., 2010; Perez et al., 2013; Zhang et al., 2015). For example, an increase in Mn^2+^ signal intensity in dorsal CA1 and dentate gyrus of mice showing neurodegeneration and forebrain atrophy might be associated with increased presence of glial cells in the region (Perez et al., 2013) and an increase in the area of Mn^2+^ intensity was observed at the mossy fiber to CA3 synaptic terminal region following a learning (Zhang et al., 2015). In spite of these interesting results, a significant limitation of MEMRI is the neurotoxic effects of Mn^2+^ on dopamine neurons (Aschner et al., 2007), and its actions as a glutamatergic NMDA receptor blocker, both of which may interfere with its intended use of measuring neuronal activity and aging related changes in neuronal activity (Guilarte and Chen, 2007; Liu et al., 2013). Furthermore, because Mn^2+^ competes with Ca^2+^, it will have effects on Ca^2+^-dependent processes including the release of transmitter and possible synaptic plasticity (Eschenko et al., 2010). Systemic administration of Mn^2+^ may also affect overall health and chronically affect weight gain in rats (Jackson et al., 2011), thus further reducing the utility of this method for longitudinal MRI studies.

## Pharmacological MRI of Potential Cognitive Modulators

BOLD fMRI, arterial spin labeling, and superparamagnetic iron oxide nanoparticle based functional imaging of blood volume are also used for *in vivo* measurement of drug-induced brain activation. The use of these modalities initially started with administration of psychoactive substances in studies of drug abuse and addiction (Mandeville et al., 1998; Marota et al., 2000; Schwarz et al., 2003; Febo et al., 2004, 2005a,b), but over the last decade other applications, particularly the testing of modulators of cognitive function and mood has emerged. For instance, one of the key mechanisms involved in LTP is an increase in AMPA receptor-mediated synaptic currents through the insertion of AMPA receptors into the post-synaptic terminal (Luscher et al., 2000). Drugs that enhance AMPA mediated effects can thus be considered to be potential targets for modulating memory through a well-defined synaptic mechanism. Administration of an AMPA receptor agonist (LY404187) increased BOLD activation largely in the dorsal hippocampus and septum and this was blocked by pretreatment with the AMPA/kainite antagonist LY293558 (Jones et al., 2005). This is consistent with previous work with the same AMPA agonist compound showing increases in cerebral metabolic rates for glucose and c-fos expression in the same regions (Fowler et al., 2004). Cholinergic modulation in the brain has also been assessed using drugs that activate muscarinic and nicotinic receptors (Hoff et al., 2010, 2011; Haley et al., 2011; Bruijnzeel et al., 2014). Experiments focusing on the cholinergic system show pronounced thalamocortical activation, which is very low in studies examining AMPA receptor activation. This shows the capacity of combining pharmacology and fMRI to distinguish among these two drug classes acting through different receptor systems. Bruijnzeel et al. (2014) showed dose-dependent nicotine-induced BOLD activation of anesthetized rat brain, which was blocked by the general nicotine receptor antagonist mecamylamine (Bruijnzeel et al., 2014). Administration of the non-specific muscarinic receptor antagonist scopolamine to aged monkeys resulted in greater increases in hippocampal BOLD signal, but only in animals performing well on spatial maze task (Haley et al., 2011). This correlated with greater levels M1 but not M2 receptor density in greater performing than poorly performing animals.

In humans, an age-related decline in activity within the posterior brain regions including the hippocampus is associated with increased activity in the prefrontal cortex (Sperling, 2007; Kaufmann et al., 2008; Park and Reuter-Lorenz, 2009; Turner and Spreng, 2012; Lighthall et al., 2014; Tromp et al., 2015). Interestingly, inhibition of NMDA receptors in the hippocampus or prefrontal cortex drives activity in the prefrontal cortex (Jodo et al., 2005; Homayoun and Moghaddam, 2007). Furthermore, low level NMDA receptor blockade impairs hippocampal function and improves executive processes that depend on the prefrontal cortex (Guidi et al., 2015b). Chin et al. (2011) showed that ketamine, an amnesic/dissociative agent that blocks NMDA receptors produced robust activation of cortical regions and the hippocampus of awake rats. This effect was modulated by the glutamate metabotropic agonist LY379268 (Chin et al., 2011). More recent work has confirmed that ketamine increases functional interactions between brain regions involved in memory (hippocampus, areas of the limbic prefrontal cortex, and retrosplenium; Gass et al., 2014; Grimm et al., 2015). These studies illustrate the potential for pharmacological MRI to investigate brain wide activation in response to cognitive modulators.

## Diffusion Brain Imaging in Normal Aging Rats

Compared with fMRI, diffusion MRI has been applied more extensively to the study of animal models of neurodegenerative diseases. However, we will mostly focus here on dMRI studies relevant to normal cognitive aging. In diffusion MRI, directionally applied diffusion-sensitizing magnetic field gradients tag protons in slowly moving (diffusing) water molecules (in the order of 10^-3^ μm^2^/s), and thus omit water moving at faster rates (e.g., inside blood vessels, as measured in the above-cited fMRI modalities; Le Bihan et al., 2001). The fractional anisotropy (FA) index is one of the main scalars estimated from a series of diffusion-sensitized MRI images. FA has been used extensively as an indicator of underlying tissue microstructural integrity (Mori and Zhang, 2006). This value is most reliable when assessed in major white matter (WM) tracts in rodent brain at high fields, although a growing number of studies are also reporting FA for gray matter regions. FA values range from 0 to 1, with 1 indicating *high directionality* of water diffusion (anisotropic diffusion) and 0 *low directionality* (isotropic diffusion). Thus, lowest FA values are measured from cerebroventricles (because of the high mobility of unbound water molecules have in this compartment), whereas highest values are measured in WM fiber bundles, such as the corpus callosum, fimbria, internal capsule, where water molecules show a high net directionality due to the presence of highly organized barriers to diffusion formed by myelinated axonal fibers. Reductions in myelination, increases in fluid filled inter-axonal spaces, altered cellular density, local cellular inflammatory responses, and edema can all reduce FA (Peled, 2007). Reduced FA is thought to represent WM alterations in aging and pathologies of Alzheimer’s disease in humans (Bozzali et al., 2001; Teipel et al., 2010; Douaud et al., 2011, 2013). Recent evidence for a link between reduced FA and pathology was provided by studies in a mouse overexpressing microtubule associated protein tau (Sahara et al., 2014). Similar to human studies, the results demonstrate a progressive reduction of FA in WM structures of the tau-expressing mice. Changes in FA were associated with an increase in tau pathology and disorganization of unmyelinated processes. Indeed, changes in dMRI were detectable as early as 2.5 months, before the emergence of obvious overt pathology. Similar age-associated reductions in FA (and concomitant increases in diffusivity scalars, axial, radial, and mean diffusivities) have been reported in a transgenic rat model of Huntington disease (Antonsen et al., 2013), and in corpus callosum of normal aged rats (Guo et al., 2015). In spite of these correlations, the mechanistic basis for changes in FA still remains unclear.

In humans, it has been known for many years that WM content in brain shows an inverted U maturational change that peaks at middle age (45 years of age). This was first demonstrated in postmortem tissue and subsequently supported by Bartzokis et al. (2004) using MRI. The loss of WM begins early in middle-age in humans and rhesus monkeys, with a prolonged decline during aging (Makris et al., 2007; Westlye et al., 2010; Yeatman et al., 2014). In contrast, WM loss is initiated much later for chimpanzees, suggesting that older chimpanzees exhibit decreased atrophy relative to humans (Chen et al., 2013). Rodents show a age progressive change in WM similar to humans, occurring at earlier stages in the rodents lifespan, with a steep rise in FA from 0–40 days (ending at mid adolescence), and a gradual but progressive decline thereafter (Calabrese et al., 2013). Interestingly, mean diffusivity peaks earlier between 10–20 days of age and then remains stable, or shows a steady decline (Mengler et al., 2014), at least until day 80 (Calabrese et al., 2013). FA values in outer cortical layers of developing rat brain is reduced during the first 10 postnatal days (Huang et al., 2008). Therefore, diffusion MRI can distinguish between non-WM maturational changes. Thus, these represent early life maturational changes, perhaps associated with early brain development (Mengler et al., 2014). Synaptic and axonal pruning and increases in myelination of major fiber tracts account in part for these early life changes in FA and mean diffusivity (Huang et al., 2008). Compared to 3-month-old rats, 12-month-old rats show lower apparent diffusion coefficient (ADC) values in cortex (Heiland et al., 2002). ADC mapping is typically used in preclinical stroke research, with reduced gray matter ADC values indicative of early hemorrhagic events and high values indicative of progressive edematous tissue damage. Aged rats sustaining transient global ischemia also show greater reductions in ADC than young animals imaged under the same conditions (Canese et al., 1998, 2004). Hypotension-associated with a single hemorrhage event causes a greater reduction in hippocampal ADC in 18-month-old compared to 12-month-old rats (Plaschke et al., 2009). Thus, vascular events that increase in risk with age are observed to alter diffusion MRI indices of tissue integrity. While this is an area that needs further investigation, it points to the possibility of developing the diagnostic capabilities of diffusion MRI as a technique that offers tissue quantitative measures that could assess risk or vulnerability in aging brain under specific challenges. For example, age-related reduction in FA in corpus callosum is prevented in aged rats subjected to a caloric restriction (Guo et al., 2015).

## Proton Magnetic Resonance Spectroscopy

A major advantage of ultra-high field imaging (7 T and above), apart from the greater signal-to-noise, is the improved capacity to resolve or separate the chemical shift peaks of various biomolecules involved in neurotransmitter metabolism in cells (Di Costanzo et al., 2003; Moser et al., 2012). MRS, particularly involving hydrogen (^1^H) nuclei, has been used for years to assess various chemical species in brain diseases, both neuropsychiatric and neurologic conditions (Maddock and Buonocore, 2012; Rossi and Biancheri, 2013). Imaging techniques have better spatial resolution than MRS techniques, but ^1^H-MRS offers strong complementary data because of its specificity and quantitative capabilities, which permit the assessment of tissue concentrations of distinct biologically relevant molecules and metabolic intermediates. ^1^H-MRS can detect molecular markers for neurons and glia, transmitters, and antioxidant capacity (see **Table 1**).

**Table 1:** Proton MRS markers relevant to aging, inflammation, neurodegeneration, and excitatory neurotransmission.

Molecule or metabolic intermediate	Marker for	Age-related change
NAA	Neuronal health	No change with normal aging, decreased in neurodegenerative disease
myo-inositol	Glia	Increased in normal aging, possibly as a sign of neuroinflammation
Acorbate, GSH	Oxidative stress	Generally decreased with age, indicating brain regions that are vulnerable to oxidative stress
Glutamate, GABA	Neurotransmitters	Region specific changes may reflect a shift in the balance of excitatory/inhibitory transmission

For example, *N*-acetylaspartate (NAA) and myo-inositol are measured as neuronal and glial markers, respectively (Demougeot et al., 2004; Harris et al., 2015). Following experimental ischemia, a decline in NAA is observed in the most vulnerable brain regions and correlates with cell loss (Higuchi et al., 1997; Sager et al., 2001). However, there is also evidence for a decline in NAA not linked to neuronal loss (Jenkins et al., 2000). NAA is synthesize in the mitochondria and a rapid decline in NAA following ischemia may represent impaired neuronal function, which can recover during reperfusion (Demougeot et al., 2004). Indeed, minor levels of hippocampal cell loss following mild ischemia was not associated with a change in hippocampal NAA (Galisova et al., 2014).

In Alzheimer’s disease, a decline in NAA is observed in hippocampus and posterior cingulate in association with a loss of synaptic markers and increased hyperphosphorylated tau, consistent with a neuronal loss. Interestingly, increased myo-inositol levels where more prominent in the cortex in association with amyloid-beta levels, suggesting activation of glial markers may precede neuronal loss (Murray et al., 2014; Wang et al., 2015). In this case, the increase in glial markers may be an early sign, possibly representing neuroinflammation. Indeed, APP/PS1 transgenic mice, an increase in myo-inositol precedes the decline in NAA and changes in both markers precede cognitive impairment (Chen et al., 2012).

In the case of normal aging in humans and in animal models of brain aging, an increase in glial markers, possibly as a sign of neuroinflammation, is observed in the absence of profound neuronal loss. Studies of ^1^H-MRS profiles in aging humans indicates no change or a small decrease in NAA and an increase myo-inositol, consistent with an increase in glial markers (Boumezbeur et al., 2010). Similar changes are observed in ^1^H-MRS profiles of aging non-human primates (Herndon et al., 1998; Ronen et al., 2011) and rodents (Driscoll et al., 2006; Duarte et al., 2014; Harris et al., 2014), consistent with an increase in astrocytic markers associated with chronological age. A consistent observation of aging in humans and animal models, is a persistent low level increase in serum markers of inflammation and this pro-inflammatory phenotype is thought to influence neuroinflammation, the activation glial cells, astrocytes and microglia, and may contribute to age-related cognitive decline (Rafnsson et al., 2007; Gimeno et al., 2008; Bettcher et al., 2012; Scheinert et al., 2015). Studies that employ ^1^H-MRS and correlate brain levels of myo-inositol with measures of systemic inflammation indicate a positive relationship (Eagan et al., 2012; Schneider et al., 2012). Interestingly, just as with neurodegenerative disease and aging, not all brain regions are equally vulnerable to the effects of systemic inflammation (Semmler et al., 2005; Silverman et al., 2014; Scheinert et al., 2015) and regional markers of inflammation, including levels of myo-inositol correlate with behavioral changes (Schneider et al., 2012; Scheinert et al., 2015).

One important goal for the neurobiology of aging is to understand regional differences in neuronal vulnerability to aging (Jackson et al., 2009; Wang and Michaelis, 2010; Zeier et al., 2011). Total choline levels may reflect membrane turn over including demyelination and inflammation and brain levels increase with age in humans and animal models (Duarte et al., 2014; Harris et al., 2014). However, it is unclear whether the age-related increase in total choline is due to membrane turnover or linked to functional changes including cognitive decline (Charlton et al., 2007). Thus, animal studies could provide a rich source for hypotheses concerning the cellular and molecular constituents of total choline measures, as well as a test of regional vulnerability. Oxidative stress increases with advancing age and oxidative damage may contribute to neuronal death associated with neurodegenerative disease. Recent work indicates that the oxidation–reduction (redox) status of neurons underlies senescent physiology including impaired synaptic plasticity and the emergence of cognitive impairment (Bodhinathan et al., 2010a,b; Kumar and Foster, 2013; Lee et al., 2014; Guidi et al., 2015a). ^1^H-MRS can be employed to examine redox status by measuring the level of antioxidant molecules, ascorbate and glutathione (GSH). GSH is mainly observed in astrocytes (Slivka et al., 1987; Keelan et al., 2001) and an increase in GHS could indicate activation of astrocytes. Alternatively, a decline in GHS could signal an increase in oxidative stress. In rats, maturation is associated with an increase in the level of GSH in the prefrontal cortex and prenatal immune activation interferes with an inability to increase GSH levels (Vernon et al., 2015). In general, aging is associated with a decline in antioxidant molecules in the brain. Moreover, the decline is regionally selective and can vary by gender (Terpstra et al., 2006; Emir et al., 2011; Duarte et al., 2014; Harris et al., 2014), which provides grist for hypotheses concerning vulnerability to aging and neurodegenerative disease.

Finally, ^1^H-MRS can detect the level of certain transmitters. The most common measures are for glutamate, aspartate, and gamma-aminobutyric acid (GABA). Changes in transmitter levels are observed in several animal models of neurological diseases (Biedermann et al., 2012; Bagga et al., 2013; Berthet et al., 2014; Santin et al., 2014). In some cases, the animal models exhibit a good correspondence with the human condition. For example, altered levels of glutamate are observed in the prefrontal cortex of schizophrenia patients and animal models of schizophrenia (Iltis et al., 2009; Maddock and Buonocore, 2012; Puhl et al., 2015). Similarly, a decrease in prefrontal cortex glutamate is observed in depressed patients and in animal models of stress (Knox et al., 2010; Hemanth Kumar et al., 2012; Drouet et al., 2015). Examination of transmitters over time may give clues to mechanisms and how processes change over time. A decline in both glutamate and GABA show a progressive decline in animal models of Alzheimer’s disease (Nilsen et al., 2012) and a differential decline in glutamate and GABA is observed during the development of temporal lobe epilepsy (van der Hel et al., 2013). An increase in glutamatergic transmission may contribute to the development of Parkinson’s disease. MRS studies examining glutamate levels in the striatum of humans with Parkinson’s disease do not agree possibly due to the etiology, stage of the disease, or pharmacological intervention (Kickler et al., 2007; Griffith et al., 2008; Modrego et al., 2011). Similarly, in animal models differences in glutamate levels may depend on the etiology or the animal model (Delli Pizzi et al., 2013). For example, treatment with 1-methyl-4-phenyl-1,2,3,6-tetrahydropyridine decreased glutamate levels in felines (Podell et al., 2003), no change in canines (Choi et al., 2011), and increased glutamate levels in mice (Chassain et al., 2008, 2013), Even when examined in the same animal model differences may arise associated with the power of the magnet (Kickler et al., 2009; Coune et al., 2013).

Regional differences in transmitter levels are associated with aging in humans (Grachev et al., 2001; Gao et al., 2013; Zahr et al., 2013; Riese et al., 2015). In male rats, aspartate and glutamate exhibited a decline with in specific brain regions, which may reflect a shift in the balance of excitatory and inhibitory transmission (Harris et al., 2014). In contrast, measures of both GABA and glutamate or aspartate declined in an aging male and female mice (Duarte et al., 2014). Future studies will need to investigate these discrepancies which may include regions examined and sex differences in glutamate over the course of aging (Zahr et al., 2013), as well as variability associated with variability in cognitive decline.

## Concluding Remarks

One of the key aspects of aging brain is a gradual loss of memory to the point where this affects the individuals’ normal daily activities (Gauthier et al., 2006). In normal aging cognitive impairment can be progressive, and while not necessarily entailing a severe loss of neurons, as in neurodegenerative diseases, the animal literature supports alterations in neuronal activity (Kumar and Foster, 2007; Watson et al., 2012; Foster et al., 2016). Indeed, these changes may involve alterations in excitatory/inhibitory balance, changes in synaptic proteins and intracellular signaling mechanisms, and spine density and morphology (Foster et al., 1996, 2000, 2012, 2016; Foster and Norris, 1997; Foster and Dumas, 2001; Blalock et al., 2003; Foster, 2007, 2012; Kumar and Foster, 2007, 2013; Kumar et al., 2009; Dumitriu et al., 2010; Guidi et al., 2015a,b). Translating these cellular mechanisms of animal aging models to human aging is a difficult challenge and it may be possible that preclinical imaging and spectroscopy studies could serve a role in this task. We have reviewed different MRI modalities used in primate and rodent models to characterize functional activation in hippocampal and prefrontal memory networks, anatomical changes and their correlations with cognitive decline, changes in neurovascular coupling with aging, and biochemical alterations relevant to aging. Results from the different MR modalities presented here can be enhanced by combining these with invasive *in vivo* and *ex vivo* approaches to determine their relationship to changes at the synaptic, proteomic, and genetic levels, for an integrative assessment of brain aging and reduced cognitive capacity.

## Author Contributions

MF and TF contributed equally to the outline, drafting and editing of the present manuscript.

## Conflict of Interest Statement

The authors declare that the research was conducted in the absence of any commercial or financial relationships that could be construed as a potential conflict of interest.
